# Bacterial Leakage of Mineral Trioxide Aggregates and Portland Cement

**Published:** 2006-10-01

**Authors:** Mohammad Asnaashari, Saeed Asgary, Asef Khatami

**Affiliations:** 1 *Department of Endodontics, Dental School, Shahid Beheshti University of Medical Sciences, Tehran, Iran*; 2 *Department of Endodontics, Dental Research Center, Dental School, Shahid Beheshti University of Medical Sciences, Tehran, Iran*; 3 *General practitioner, Tehran, Iran*

**Keywords:** Bacterial Leakage, MTA, Portland Cement, Root End Filling

## Abstract

**INTRODUCTION:** The purpose of this study was to compare the ability of ProRoot mineral trioxide aggregate (PMTA) and Root mineral trioxide aggregate (RMTA) as root-end filling materials, and Portland cement (PC) to prevent bacterial leakage through filled root end cavities.

**MATERIALS AND METHODS:** Fifty-one extracted human single rooted teeth were cleaned and shaped using a step back technique. The root-ends were resected and a 4 mm deep root-end preparation was made with fissure bur. The teeth were randomly divided into three experimental groups (n=15) and a further six teeth served as controls. Three root-end cavities were filled with gutta-percha without a root canal sealer (positive control) and three remaining were filled with sticky wax, covered with two layers of nail polish (negative control). Root-end cavities in each experimental group were filled with PMTA, RMTA or PC. After attaching the teeth to plastic caps of 9 ml vials, the teeth and the caps were sterilized using Gamma ray. Then the caps with teeth were placed on the vials containing Phenol Red Lactose broth. A tenth of microliter of Tripticase Soy broth containing Staphylococcus Epidermidis (SE) was placed into the root canals of the teeth. Every 48 hours inoculation of 0.1 ml of the SE broth culture into each root canal was performed.

**RESULTS:** All positive controls leaked within 3 days, none of the negative controls leaked. Bacterial leakage occurred in 33% of samples in the PMTA group and in 40% of samples in RMTA and PC groups. The results indicated no statistical difference between three test materials after 35 days.

**CONCLUSION:** It was concluded that PMTA, RMTA and PC demonstrated a similar ability to seal root end cavities.

## INTRODUCTION

When a non-surgical root canal therapy fails or is contraindicated, periradicular surgery with a root-end filling material is necessary. The most important factor in determining the success of a periradicular surgery is the efficiency of the apical seal. The apical seal inhibits leakage of residual irritants from the root canal into the periradicular tissues. Periradicular lesions of endodontic origin have been associated with the presence of bacteria and their by-products in an infected root canal system (1). Root-end filling materials seal the content of the canal preventing egress of bacteria or toxic materials into the periradicular tissues.

The sealing ability of root-end filling materials has been assessed by different methods such as dye or bacterial penetration, electrical methods, fluid filtration technique, radioisotope tracing, and marginal adaptation by SEM. Dye penetration and Radioisotope techniques have been the most frequently used methods to evaluate the sealing ability of various root-end filling materials (2).

Numerous materials have been recommended to be used as root-end filling materials such as amalgam, gutta-percha, composite resins, glass ionomers, mineral trioxide aggregate (MTA), and some other restorative materials.

Sealing ability of these materials have been compared in many studies, and most of them have shown that ProRoot MTA (PMTA) have significantly less leakage than other root-end filling materials, and is considered as a high quality root-end filling material (3-4).

Recently a new similar material, Root MTA (RMTA), has been developed in Iran, which is made of the same components of PMTA (5). In various in vivo and in vitro studies, it has been proved that RMTA has so many similarities with PMTA (6-8).

Newly the chemical, physical and biological properties of Portland cement (PC) have been analyzed. It was shown that PC contained the same principal chemical elements as MTA, except for bismuth oxide (9-10). Wucherpfenning & Green reported that MTA and PC were almost macroscopically and microscopically identical, when evaluated by X-ray diffraction analysis (11). Other researchers have also reported that MTA and PC had similar properties (12-13).

The purpose of the present *in vitro* study was to compare the sealing ability of PMTA, RMTA and PC in extracted human single rooted teeth, using a bacterial leakage model.

## MATERIALS AND METHODS

Fifty-one extracted human single rooted teeth were selected. After initial radiographs, standard access cavities were prepared. Using the step back technique, all teeth were instrumented to a #70 k-files (Mani, Japan). During cleaning and shaping, copious irrigation was performed with sodium hypochlorite solution (2.5%). At 3 mm from the apical end of the root, an apical resection at 45 degrees to the long axis of the tooth was made under water spray with a fissure bur (D&Z, Germany). Standardized class I root-end preparation were made to a depth of 4 mm using 008 fissure burs (D&Z, Germany) under spray water. The prepared teeth were randomly divided into three experimental groups of 15 teeth each, and two control groups of 3 teeth each. Before placing the root end fillings, the cavities were dried using paper points (Aryadent, Iran).

**Figure 1:**
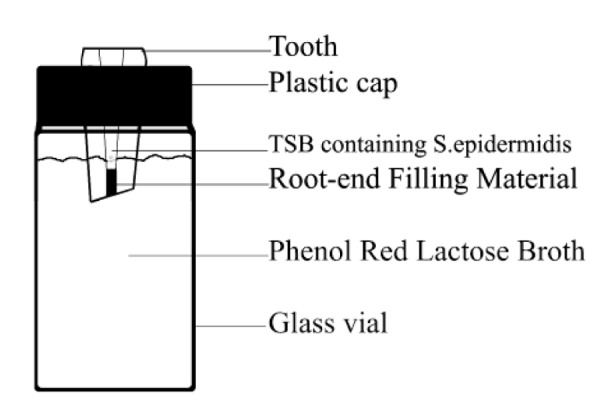
Diagram representing the microbial test system used to determine bacterial leakage

The root end filling materials were inserted as the following manner: PMTA in group 1 (ProRoot MTA, Dentsply, Tulsa, OK, USA), RMTA in group 2 (Root MTA, Salamifar Co, Tehran, Iran), and PC in group 3 (Simane Abyek, Qazvin, Iran) were prepared according to the manufacturer’s directions and placed into the root end cavities. Three root-end cavities filled with sticky wax and covered with two layers of nail polish, served as negative controls. Positive controls were three root-end cavities filled with gutta-percha without sealer. Two layers of nail polish were applied to the external surfaces of all roots to prevent bacterial leakage through the lateral and or accessory canals.

Nine milliliter vials (Hepatitis vaccine vials) were used to suspend the prepared teeth. The snap on caps were punctured. The teeth were placed into holes of the caps and sticky wax was used to seal the gaps between teeth and caps. This complex was sterilized using Gamma ray. Phenol Red Lactose (PRL) broth was placed in each vial to a level of 2 to 3 mm above the resected root-ends (Figure 1). A sterile Insulin syringe was used to inoculate 0.1 ml of an over night broth culture of SE into the root canal of each tooth via the coronal access cavity preparation. Any leakage of the SE from the root canals into the PRL broth would become evident when the color of the broth changes from red to yellow as a result of acid production from bacteria growth. The entire apparatus was then placed into an incubator, maintained at a constant 37^°^C. After sterilizing the teeth with Gamma ray, all of the works and procedures were performed under a biological safety hood with strict adherence to aseptic technique. Inoculation of SE was performed every 48 hours. The PRL broth vials were monitored every day for color changes. If a color change occurred, a sample of yellow medium was plated on TSB and then on blood agar with presence of Novobiocine antibiogram disks, and incubated at 37^°^C to confirm the bacterial presence as SE. The experiment was run for 35 days.

A Kruskal Wallis and Chi-Square tests were used to determine the statistical differences between various groups.

## RESULTS

All specimens of the positive control group showed color change in the PRL broth within 3 days of incubation. No evidence of color change in the PRL broth occurred in the negative control group during the experimental period.

Five of the 15 samples of the group 1 (PMTA) were fully contaminated within 5 to 25 days. Six of the 15 samples of the group 2 (RMTA) were fully contaminated within 5 to 27 days. Six of the 15 samples of the group 3 (PC) were fully contaminated within 5 to 30 days. In all cases when color changes occurred, the bacteriological tests showed that the bacteria present in the growth medium was SE.

The data obtained were statistically analysed with the Kruskal Wallis and Chi-Square tests which showed no statistically significant difference between the three experimental materials (P>0.05).

## DISCUSSION

Dye leakage studies have been used for many years to evaluate the sealing ability of endodontic materials. Dye penetration studies tend to overestimate leakage as the size of dye molecule is smaller than bacteria (2). Despite the ease of use, dye leakage studies have several disadvantages: a) like radioisotope traces, the molecular size of most dye particles is smaller than bacteria, so it causes dissimilar leakage patterns. b) Most dye leakage studies have measured the degree of leakage in one plane, making it impossible to evaluate the total leakage, and c) compared with clinical conditions, in vitro dye studies are static and do not reflect the dynamic interactions between the root canals and periradicular tissues (15).

Studies using bacterial cultures have been used widely to test the sealing ability of endodontic materials and may be considered to have more biological relevance than dye leakage tests. Obviously, bacterial leakage studies have much resemblance with oral environment and condition, bacterial flora of the mouth, and also leakage pattern rather than other techniques (16).

In this study we evaluated the sealing ability of a 4 mm thickness of PMTA, RMTA (as root-end filling materials), and PC (as an alternative material) using a bacterial leakage model. Positive and negative controls responded as expected. The entire positive control group exhibited a color change which indicates that a root canal sealer is needed to improve gutta-percha as a root end filling material.

Incubation of bacteria started 48 hours after placing the plastic caps and teeth, on the PRL broth vials. The absence of color changes in this period of time indicates that we could provide a contamination free environment, for the test of root end filled samples.

Considering that PMTA contains the same principal chemical elements as PC (9-13), as well as RMTA (5), they probably have a similar mechanism of action and thus the results of biological investigation are similar.

It was shown that the mechanism of action of PMTA and PC were similar. Both materials contain calcium oxide that forms calcium hydroxide when mixed with water. The reaction of the calcium hydroxide and the carbon dioxide from the pulp tissue produces calcite crystals (12,17). These findings strongly support the role of calcite crystals and fibronectin as an initiating step in the formation of a hard tissue barrier.

Shokouhinejad et al. compared the sealing ability of White and Gray PMTA, RMTA, and PC as root-end fillings using bacterial leakage method and reported comparable results of the test materials (8). Similar finding was also shown by other researchers (7,18).

The results of the present study support those described previously (7-13,17-18) suggesting that PMTA, RMTA and Portland cement are almost similar.

Based on these results, it seems that there is no significant difference between PMTA, RMTA and PC and considering all other characteristics of these three materials, it is possible to replace and substitute PMTA, with RMTA and even Portland cement. But just because the Portland cement is a non medical product, use of this industrial material is not recommended.

## CONCLUSION

Under the conditions of this *in vitro* study, the bacterial leakage patterns of Pro-Root mineral trioxide aggregate, Root mineral trioxide aggregate, and Portland cement in root-end fillings were similar over a period of 35 days.
